# Return to Work After Adjuvant Chemotherapy for Colon Cancer: A Retrospective Study With Exploratory Occupational Analysis

**DOI:** 10.7759/cureus.116522

**Published:** 2026-09-19

**Authors:** Vanessa Duarte Branco, Francisca Liz, Marta Orey, Pedro Frazão, Maria Abreu, Mariana Malheiro, Susana Santos Amador, Débora Cardoso, Ana Martins

**Affiliations:** 1 Medical Oncology, São Francisco Xavier Hospital, Western Lisbon Local Health Unit, Lisbon, PRT; 2 Medical Oncology, CUF Tejo Hospital, Lisbon, PRT; 3 Medical Oncology, Comprehensive Health Research Centre, NOVA Medical School, NOVA University, Lisbon, PRT; 4 Occupational Health Unit, Interprev, Lisbon, PRT

**Keywords:** adjuvant chemotherapy, cancer survivorship, colon cancer, occupational health, occupational reintegration, return to work, workplace autonomy

## Abstract

Background

Return to work is an important survivorship outcome for working-age cancer survivors. Still, occupational trajectories after adjuvant chemotherapy for colon cancer are not always systematically documented in routine oncology care. This study aimed to describe documented occupational trajectories after adjuvant chemotherapy for colon cancer and to explore occupation-level physical demand and break/restroom autonomy as descriptive occupational dimensions.

Methods

This single-centre retrospective observational study was based exclusively on routinely collected clinical records. Working-age patients with histologically confirmed colon adenocarcinoma, active work attachment before diagnosis, and completion of adjuvant chemotherapy between January 2024 and December 2025 were included. No questionnaire-derived data, Work Ability Index scores, or quality-of-life instruments were used. The primary analysis was descriptive and focused on ascertainable occupational trajectories documented in routine oncology records. Patients with return to work mentioned without an exact date, insufficient occupational information, disability retirement, unemployment, or documented recurrence before return were not treated as uniformly right-censored observations.

Results

Among 62 eligible patients, two did not interrupt work during treatment and were described separately. Among the 60 patients who interrupted work, 34 had a documented return-to-work date. The observed median time from completion of adjuvant chemotherapy to documented dated return among these 34 patients was 115 days. Eight additional patients had return to work mentioned in the records without an exact date. Among the 26 patients without a dated return-to-work event, three were documented as still on medical leave or without return at the last occupational note, four entered disability retirement before documented return, eight were unemployed without documented return, eight had undated return mentioned, two had insufficient post-chemotherapy occupational information, and one had documented recurrence before return. Occupational coding was feasible in 45 patients; 26 of these had a documented return date. In descriptive exploratory analyses, 22 of 34 patients in higher-autonomy occupations and four of 11 patients in low-autonomy occupations had a documented dated return-to-work event.

Conclusions

Occupational trajectories documented in routine oncology records after adjuvant chemotherapy for colon cancer were heterogeneous and incompletely captured. The absence of a dated return-to-work entry should not be interpreted as confirmed non-return in all patients. The exploratory occupational analysis did not establish physical demand or break/restroom autonomy as determinants of return to work. These findings support more systematic documentation of occupational status in survivorship care and prospective studies using validated individual-level occupational measures.

## Introduction

Improvements in cancer survival have shifted attention from disease control alone to broader survivorship outcomes, including functional recovery, social participation, financial security, and quality of life. Among working-age survivors, return to work is a particularly relevant marker of recovery because it reflects the interaction between health status, functional capacity, job demands, workplace support, and social protection systems [1-4].

For colon cancer survivors, occupational reintegration may be complex. Studies in colorectal cancer populations show that work participation and work disability may be influenced by treatment burden, chemotherapy, persistent symptoms, disease stage, sociodemographic circumstances, and occupational context [5-11]. Surgery, adjuvant chemotherapy, chemotherapy-induced neuropathy, bowel dysfunction, fatigue, pain, and psychological distress can persist beyond completion of treatment and may affect confidence and feasibility of returning to previous occupational roles [8,10,11]. Qualitative evidence further suggests that return to work is shaped by employer understanding, access to accommodations, gradual or adapted return, stigma, financial pressure, family circumstances, and the need to manage bowel control in the workplace [12,13].

Return to work should not be interpreted as a simple binary endpoint. Some survivors continue working throughout treatment, others return gradually or with adaptations, some return and subsequently stop, and others remain on medical leave, become unemployed, or progress to disability retirement. This complexity supports the need to describe occupational trajectories rather than only whether a patient has ever returned to work [4,12,13].

Most research on employment after cancer has focused on clinical and sociodemographic determinants, while the concrete characteristics of the job itself remain less consistently captured [4,8,14,15]. For gastrointestinal cancers, this may be particularly relevant: physical demands, inflexible schedules, limited autonomy to pause tasks, or restricted access to restroom facilities may make occupational reintegration more difficult, even among clinically disease-free patients.

Evidence on return-to-work outcomes among colon cancer survivors remains limited, especially in Portuguese clinical settings. In addition, occupational status is not always systematically documented in routine oncology follow-up, which may limit understanding of work-related survivorship outcomes in real-world practice. This study therefore aimed to describe occupational trajectories and dated return-to-work events documented in routine clinical records after adjuvant chemotherapy for colon cancer among working-age patients with active work attachment before diagnosis. As an exploratory, hypothesis-generating objective, we also described occupation-level physical demand and autonomy for breaks/restroom access in relation to documented occupational trajectories.

## Materials and methods

Study design and setting

This was a single-centre retrospective observational study based exclusively on routinely collected clinical records. The study was conducted in a hospital-based oncology setting in Portugal. Reporting was aligned with the Strengthening the Reporting of Observational Studies in Epidemiology guidance for observational studies [16].

No patient-reported questionnaires, interview-derived data, Work Ability Index scores, or quality-of-life instruments were used in this version of the study. All variables analysed were extracted from clinical records or structured clinical data sources generated during routine care.

Clinical records were reviewed through July 8, 2026, the latest occupational-status documentation date included in the database; this was used as the database cut-off date. No clinical or occupational information recorded after this date was included in the analysis. Occupational follow-up was defined from the date of completion of adjuvant chemotherapy to the last clinical documentation of occupational status available before database closure. Occupational status was not collected at predefined study visits; it was ascertained from routine oncology follow-up notes, clinical correspondence, structured chart-review fields, and any relevant administrative or social documentation available in the clinical record.

Participants

Potentially eligible patients were identified through medical record review. The inclusion and exclusion criteria are summarized in Table 1.

**Table 1:** Inclusion and Exclusion Criteria Note: Temporary sickness leave after diagnosis or during follow-up was not an exclusion criterion if active work attachment before diagnosis was confirmed.

Criterion Type	Criterion
Inclusion	Age 18-66 years.
Inclusion	Histologically confirmed colon adenocarcinoma.
Inclusion	Adjuvant chemotherapy completed between Jan 1, 2024, and Dec 31, 2025.
Inclusion	Employment, self-employment, or active work attachment before diagnosis.
Inclusion	Occupational status and follow-up documented in routine clinical records.
Exclusion	Retired before diagnosis.
Exclusion	Permanent disability retirement before diagnosis.
Exclusion	Unemployed without active work attachment before diagnosis.
Exclusion	No previous work history documented before diagnosis.
Exclusion	Diagnosis other than colon adenocarcinoma, including rectal cancer.
Exclusion	Insufficient documentation to confirm active work attachment or eligibility.

For the purposes of this study, active work attachment referred to paid employment, self-employment, or an employment relationship to which the patient could potentially return after cancer-related work interruption. Patients who were on temporary sickness leave after diagnosis or during follow-up were not excluded, provided that active work attachment before diagnosis was documented. Patients were excluded if they had retired before diagnosis, had permanent disability retirement before diagnosis, were unemployed without active work attachment before diagnosis, had no previous work history documented, or lacked sufficient documentation to confirm eligibility.

Ethical considerations

According to institutional policy, formal ethics approval was not required for this retrospective analysis of anonymised data routinely collected as part of clinical care. The study was conducted in accordance with the principles of the Declaration of Helsinki and applicable data protection regulations. No identifying patient information is included in this manuscript.

Data sources and variables

Clinical and occupational variables were extracted from medical records and structured case-report forms. Variables included age, sex, diagnosis, treatment characteristics, date of completion of adjuvant chemotherapy, treatment duration, treatment-related sequelae recorded in clinical notes, professional situation before diagnosis, professional situation at last documentation, date and type of return to work when documented, date of last clinical documentation of occupational status, and alternative occupational or clinical trajectories such as medical leave, unemployment, disability retirement, subsequent work cessation, or documented recurrence before return to work.

Treatment-related sequelae were coded when explicitly documented in clinical notes. Absence of documentation was not interpreted as absence of symptoms unless the record clearly stated that no sequelae were present.

Operational definitions

Completion of main curative-intent treatment was defined as the date of completion of adjuvant chemotherapy. This date was selected as the reference point because the study aimed to describe occupational outcomes after systemic adjuvant treatment, at the transition to survivorship follow-up. The study was not designed to estimate total duration of work absence from diagnosis, surgery, start of chemotherapy, or initiation of medical leave.

Documented dated return to work was defined as the first recorded date on which the patient resumed paid work after cancer-related work interruption. Return could be full-time, part-time, or adapted according to clinical documentation.

Patients who continued working throughout treatment were classified as having no work interruption and were not considered to have a return-to-work event, because no work absence followed by return occurred.

Patients with return to work mentioned in the records but without an exact date were classified separately as having documented undated return. These patients were not treated as right-censored observations and were not included in analyses requiring an exact event date.

Patients explicitly documented as still on medical leave or without return at a known occupational follow-up date were classified as documented non-return at last occupational documentation. Patients who entered disability retirement before any documented return were classified separately as disability retirement after treatment. Patients who were unemployed without documented return were classified as unemployed without documented return. Patients with insufficient occupational information after completion of chemotherapy were classified as having insufficient post-chemotherapy occupational information. One patient with documented recurrence before return to work was classified separately as recurrence before return.

Because valid event dates or valid event-free follow-up could not be established for all patients without a dated return-to-work event, these categories were not treated uniformly as ordinary right-censored observations. The primary analysis was therefore descriptive and focused on ascertainable occupational trajectories documented in routine clinical records.

Exploratory occupational coding

When occupational title was documented, each distinct occupation was coded using two ordinal three-level exposure scales developed for this exploratory analysis. These dimensions were selected a priori because persistent fatigue, neuropathy, pain, reduced physical capacity, bowel dysfunction, and the need for unscheduled pauses may plausibly influence the feasibility of returning to work after colon cancer treatment.

Scale A assessed physical demand: low demand for predominantly sedentary or administrative work, intermediate demand for mixed standing/walking or intermittent manual tasks, and high demand for physically demanding work involving prolonged standing, lifting, or sustained manual activity.

Scale B assessed autonomy of breaks and restroom access: high autonomy when the worker could usually pause tasks and access a restroom without constraint, intermediate autonomy when pauses were possible but dependent on workflow, scheduling, or colleague cover, and low autonomy when unscheduled pauses or immediate restroom access were substantially constrained by the job structure.

Examples of lower physical-demand occupations included desk-based administrative or professional roles; intermediate physical-demand occupations included mixed roles involving standing, walking, or intermittent manual tasks; and high physical-demand occupations included roles requiring sustained manual activity, lifting, or prolonged standing. For break/restroom autonomy, high-autonomy jobs were those in which the worker could usually interrupt tasks without immediate replacement; intermediate-autonomy jobs were dependent on workflow or cover; and low-autonomy jobs were those in which unscheduled pauses could substantially disrupt core tasks or require immediate replacement.

In the occupational subgroup (n=45), physical-demand categories were distributed as follows: level 1, low physical demand, n=29; level 2, intermediate physical demand, n=12; and level 3, high physical demand, n=4. Break/restroom-autonomy categories were distributed as follows: level 1, high autonomy, n=16; level 2, intermediate autonomy, n=18; and level 3, low autonomy, n=11.

Two physicians independently coded occupations using predefined operational criteria. The coding sheet included occupational title and job information only and did not include return-to-work outcomes. The coders were therefore blinded to documented return-to-work status during initial classification. Inter-rater agreement was substantial for the physical-demand scale (Cohen's kappa 0.62; linear weighted kappa 0.71) and moderate for the break/restroom-autonomy scale (Cohen's kappa 0.44; linear weighted kappa 0.57). Discrepancies were resolved by consensus.

For descriptive analysis, the break/restroom-autonomy scale was dichotomised as low autonomy versus higher autonomy (intermediate/high combined). This dichotomisation was prespecified for the exploratory analysis because the low-autonomy category represented the most clinically relevant contrast - substantial constraint on unscheduled pauses or immediate restroom access - and because the sample size did not support stable estimates across three separate autonomy categories. Because the break/restroom-autonomy scale was not externally validated and occupational title may not capture individual workplace conditions, all occupational analyses were treated as hypothesis-generating only.

Outcomes

The primary outcome was the documented occupational trajectory after adjuvant chemotherapy among patients with active work attachment before diagnosis. Occupational trajectories included documented dated return to work, documented undated return to work, no work interruption, medical leave or no return at last occupational documentation, unemployment without documented return, disability retirement after treatment, insufficient post-chemotherapy occupational information, and documented recurrence before return to work.

Secondary descriptive outcomes included type of return when documented, sustained work participation at last documentation, subsequent work cessation after first return, disability retirement after treatment, and completeness of occupational documentation.

Exploratory occupational outcomes included the distribution of documented occupational trajectories according to occupation-level physical demand and break/restroom autonomy.

Statistical analysis

Descriptive statistics were used to characterise the sample. Continuous variables were summarised using medians and interquartile ranges and means with standard deviations when informative. Categorical variables were summarised using frequencies and percentages. Wilson 95% confidence intervals were used for key proportions. Missing data were handled by available-case analysis; no imputation was performed.

The primary analysis was descriptive. Documented occupational trajectories were summarised using ascertainable categories derived from routine clinical records. Patients with documented dated return, documented undated return, medical leave/no return, unemployment, disability retirement, insufficient occupational information, and recurrence before return were presented as separate categories rather than analysed as a single censored group.

Because several patients had return to work mentioned without an exact date, insufficient occupational information, disability retirement, unemployment, or documented recurrence before return, valid event dates or valid event-free follow-up could not be established for all patients. Kaplan-Meier estimates were therefore not retained as the primary analytical framework and were not used as the main basis for interpretation.

Among patients with a documented return-to-work date, time from completion of adjuvant chemotherapy to return was summarised descriptively. Patients without an exact return date were not included in analyses requiring a dated event.

Occupational analyses were descriptive and exploratory. Patients with missing or non-codable occupational titles were excluded only from the occupational subgroup analysis, not from the main descriptive analysis. Because of the modest sample size, small occupational subgroups, incomplete or undated occupational documentation, and the non-causal objective of the study, no multivariable model was fitted. Analyses were performed in Python using the lifelines package, version 0.30 (Python Software Foundation, Wilmington, USA).

## Results

Participant flow and baseline characteristics

Ninety-five patients aged 18-66 years with histologically confirmed colon adenocarcinoma who completed adjuvant chemotherapy between January 2024 and December 2025 were initially identified. Twelve were excluded because they had retired before diagnosis and 20 because they had no documented employment relationship or active work attachment before diagnosis. Of 63 patients potentially eligible for detailed retrospective review, one was excluded after data review because active work attachment before diagnosis could not be confirmed. The analytical sample therefore comprised 62 patients.

Two patients did not interrupt work during treatment and were described separately. The main descriptive occupational-outcome population comprised 60 patients who experienced cancer-related work interruption. Participant flow and baseline characteristics are shown in Table 2.

**Table 2:** Participant Flow and Baseline Characteristics Percentages are calculated using the analytical sample as the denominator unless otherwise specified.

Characteristic	Value
Initially identified	95
Excluded before detailed review: retired before diagnosis	12
Excluded before detailed review: no employment relationship or active work attachment before diagnosis	20
Potentially eligible for detailed retrospective review	63
Excluded after data review: active work attachment before diagnosis not confirmed	1
Analytical sample	62
No work interruption during treatment; described separately	2
Main descriptive occupational-outcome population	60
Age, years, median (interquartile range)	59.0 (56.0-62.0)
Age, years, mean (standard deviation)	58.1 (5.4)
Female sex	27 (43.5%)
Male sex	35 (56.5%)
Employed at last documentation	36 (58.1%)
Medical leave at last documentation	11 (17.7%)
Unemployed at last documentation	9 (14.5%)
Disability retirement after treatment	6 (9.7%)
Colon adenocarcinoma	62 (100.0%)
Surgery and adjuvant chemotherapy	62 (100.0%)
Treatment duration, months, median (interquartile range)	6.0 (3.0-6.0)
Treatment-related sequelae documented	13 (21.0%)

Documented occupational trajectories after work interruption

Among the 60 patients who interrupted work during the oncological trajectory, 34 had a documented return-to-work date (56.7%; 95% confidence interval: 44.1-68.4). The observed median time from completion of adjuvant chemotherapy to documented return among these 34 patients was 115 days (interquartile range: 91-183; range: 21-347).

The remaining 26 patients did not have a dated return-to-work event. This group was heterogeneous: three were explicitly documented as still on medical leave or without return at the last occupational note, four entered disability retirement before any documented return, eight were unemployed without a documented return, eight had return to work mentioned in the records but without an exact date, two had insufficient post-chemotherapy occupational information, and one had documented recurrence before return to work.

Therefore, absence of a dated return-to-work entry was not interpreted as confirmed non-return in all cases. Because valid event dates or valid event-free follow-up could not be established for all patients, the primary analysis was descriptive and focused on ascertainable occupational trajectories documented in routine clinical records.

Overall, 42 of 60 patients had return to work documented either with an exact date or mentioned without an exact date. Among the 34 patients with documented return to work, three had full return, 13 had part-time return, and 18 had adapted return. Documented occupational trajectories are summarized in Table 3.

**Table 3:** Documented Occupational Trajectories After Work Interruption CI: confidence interval; IQR: interquartile range. Confidence intervals for key proportions were calculated using the Wilson method. Patients without a dated return-to-work event represent a heterogeneous group and were not treated as uniformly right-censored observations.

Measure	Value	Population or Notes
Patients with cancer-related work interruption	60	Main descriptive occupational-outcome population
Documented dated return to work	34/60 (56.7%; 95% CI: 44.1-68.4)	Dated first return documented in clinical records
Documented return to work mentioned, dated or undated	42/60 (70.0%)	Includes dated return and return mentioned without exact date
Observed time to return among dated events, days, median (IQR)	115.0 (91.0-183.0)	Among 34 patients with documented dated return
Observed time to return among dated events, days, range	21-347	Among 34 patients with documented dated return
Full return among dated return events	3/34 (8.8%)	Among 34 dated return events
Part-time return among dated return events	13/34 (38.2%)	Among 34 dated return events
Adapted return among dated return events	18/34 (52.9%)	Among 34 dated return events
No dated return-to-work event	26/60 (43.3%; 95% CI: 31.6-55.9)	Heterogeneous category; not equivalent to confirmed non-return
Still on medical leave or no return at last occupational note	3/26 (11.5%)	Among patients without a dated return
Disability retirement before documented return	4/26 (15.4%)	Alternative/competing occupational outcome
Unemployed without documented return	8/26 (30.8%)	Occupational status documented, but no return recorded
Return mentioned without exact date	8/26 (30.8%)	Not included as dated event
Insufficient post-chemotherapy occupational information	2/26 (7.7%)	Not treated as valid right-censoring
Documented recurrence before return to work	1/26 (3.8%)	Alternative/competing clinical outcome

Exploratory occupational analysis

Occupational coding was feasible in 45 patients within the main descriptive occupational-outcome population. Fifteen patients did not have sufficiently detailed occupational information for coding and were excluded from occupational analyses only; no imputation was performed. In the codable subgroup, 26 patients had a documented dated return to work.

For physical demand, 29 patients were classified as level 1, 12 as level 2, and four as level 3. For break/restroom autonomy, 16 patients were classified as level 1, 18 as level 2, and 11 as level 3.

In the exploratory descriptive autonomy contrast, 22 of 34 patients in higher-autonomy occupations had a documented dated return-to-work event, compared with four of 11 patients in low-autonomy occupations. Given the small subgroup size, incomplete occupational documentation, lack of individual workplace assessment, and absence of adjustment for clinical or socioeconomic confounders, this finding should be interpreted only as hypothesis-generating.

Exploratory occupational findings are presented in Table 4.

**Table 4:** Exploratory Occupational Description by Physical Demand and Break/Restroom Autonomy Note: Occupational analyses were descriptive and hypothesis-generating. The occupational scales were study-specific, non-validated, and applied at the occupation level rather than through individual workplace assessment. No multivariable model was fitted.

Analysis	Estimate	Interpretation
Occupational exposure availability	45/60 patients	Occupational title available and codable; 15 patients excluded from occupational analyses only; no imputation performed
Documented dated return-to-work events in occupational subgroup	26/45 patients	Dated return documented among patients with codable occupation
Physical-demand category counts	Level 1 n=29; level 2 n=12; level 3 n=4	Level 1 = low physical demand; level 2 = intermediate; level 3 = high
Break/restroom-autonomy category counts	Level 1 n=16; level 2 n=18; level 3 n=11	Level 1 = high autonomy; level 2 = intermediate; level 3 = low
Higher-autonomy subgroup	34 patients; 22 documented dated return-to-work events	Intermediate/high autonomy combined for exploratory descriptive contrast
Low-autonomy subgroup	11 patients; 4 documented dated return-to-work events	Small subgroup; hypothesis-generating only
Occupational scale interpretation	Descriptive only	Study-specific, non-validated, occupation-level classification
Main inference	No determinant established	Physical demand and break/restroom autonomy should not be interpreted as predictors or causal determinants

## Discussion

Main findings

This single-centre retrospective study describes occupational trajectories documented in routine oncology records among working-age patients treated with adjuvant chemotherapy for colon cancer. Among 60 patients who interrupted work during the oncological trajectory, 34 had a documented dated return to work, while eight additional patients had return to work mentioned in the records without an exact date. The remaining patients had heterogeneous documented occupational or clinical trajectories, including ongoing medical leave or no return at last occupational documentation, unemployment without documented return, disability retirement, insufficient post-chemotherapy occupational information, and documented recurrence before return to work.

These findings show that occupational outcomes after adjuvant chemotherapy for colon cancer are not adequately represented by a simple binary classification of return versus no return. They also highlight that routine oncology records may capture work-related outcomes incompletely and inconsistently. Therefore, the main contribution of this study is not to provide definitive estimates of actual return-to-work probability, but rather to describe how occupational trajectories were documented in routine clinical practice and to identify important gaps in occupational follow-up documentation.

Interpretation in the context of survivorship care

Return to work is an important dimension of survivorship because it reflects not only disease status but also functional recovery, symptom burden, social participation, financial stability, and the interaction between the patient and the workplace. In colon cancer survivors, this may be particularly relevant because treatment-related symptoms such as fatigue, neuropathy, bowel dysfunction, pain, and psychological distress may persist beyond the end of adjuvant chemotherapy and interfere with occupational functioning.

In this study, several different occupational trajectories were observed or documented: some patients did not interrupt work, some returned with an exact documented date, some had return mentioned without a date, some were on medical leave, some were unemployed, and some entered disability retirement after treatment. This heterogeneity supports the need for routine survivorship care to include systematic documentation of occupational status, type of return, work adaptations, subsequent work cessation, and barriers to sustained participation.

Importantly, the absence of a dated return-to-work entry should not be interpreted as confirmed non-return in all patients. Some patients may have returned without a precise date being recorded, while others may have had insufficient occupational follow-up in the clinical record. For this reason, the present analysis prioritised descriptive occupational trajectories over time-to-event estimates.

Occupational analysis

The exploratory occupational analysis was based on occupation-level classification of physical demand and autonomy for breaks/restroom access. These dimensions were selected because physical demands, bowel symptoms, fatigue, neuropathy, and the need for unscheduled pauses may plausibly affect the feasibility of returning to work after colon cancer treatment.

However, the occupational findings should be interpreted with substantial caution. The occupational scales were developed for this study, were not externally validated, and were applied at the level of occupational title rather than through individual workplace assessment. Although occupations were coded independently by two blinded physicians and inter-rater agreement was assessed, the analysis remained descriptive, unadjusted, and based on small subgroups.

This study did not establish physical demand or break/restroom autonomy as determinants of return to work. The observed distribution of documented return across autonomy categories may suggest that workplace flexibility and the ability to pause tasks could be relevant dimensions for future research, but this should be viewed only as hypothesis-generating. Prospective studies using validated individual-level occupational measures are needed before any conclusions can be drawn regarding the role of workplace autonomy or potential interventions.

Strengths

This study has several strengths. It focused on a relatively homogeneous clinical population: working-age patients with colon adenocarcinoma treated with surgery and adjuvant chemotherapy. It explicitly required active work attachment before diagnosis, thereby avoiding inclusion of patients for whom return to work would not be a meaningful outcome. It also distinguished several occupational trajectories, including no work interruption, documented dated return, documented undated return, medical leave, unemployment, disability retirement, and recurrence before return to work.

Another strength is the exploratory occupational framework. Although not validated externally, the use of predefined coding criteria, independent blinded coding by two physicians with complementary expertise, and reporting of inter-rater agreement improve transparency. The decision not to perform multivariable modelling was also appropriate given the modest sample size, small occupational subgroups, and incomplete occupational documentation.

Limitations

This study has important limitations. First, it was retrospective and based exclusively on routine clinical records. Occupational status was not collected prospectively at predefined time points, and documentation depended on routine clinical practice. As a result, occupational data were incomplete and heterogeneous.

Second, absence of a dated return-to-work entry cannot be interpreted as confirmed absence of return to work. Among patients without a dated return-to-work event, some had return mentioned without an exact date, some were unemployed, some entered disability retirement, some had insufficient post-chemotherapy occupational information, and one had documented recurrence before return to work. These categories cannot all be treated as ordinary right-censored observations.

Third, because valid event dates or valid event-free follow-up could not be established for all patients, Kaplan-Meier estimates were not retained as the primary analytical framework and were not used as the main basis for interpretation. The primary analysis was instead reframed as a descriptive analysis of ascertainable occupational trajectories documented in routine clinical records.

Fourth, disability retirement and documented recurrence before return to work may preclude or substantially alter the possibility of occupational reintegration and should be considered alternative or competing clinical/occupational outcomes rather than ordinary non-informative censoring.

Fifth, the occupational analysis was limited by missing occupational titles, small subgroup sizes, lack of multivariable adjustment, and residual confounding. Important factors such as socioeconomic position, education, employment security, workplace accommodations, job control, comorbidities, symptom burden, and overall health status were not fully captured. The occupational scales were study-specific, non-validated, and based on occupational title rather than direct assessment of individual workplace conditions.

Finally, this was a single-centre study conducted in a Portuguese hospital-based oncology setting. Findings may not be generalizable to rectal cancer, metastatic disease, other treatment pathways, or healthcare systems with different occupational health and social protection structures.

Implications for practice and research

Despite these limitations, the study highlights the relevance of occupational outcomes in survivorship care. Routine oncology follow-up should consider documenting work status more systematically, including whether the patient is working, on medical leave, unemployed, retired due to disability, returning gradually, working with adaptations, or has stopped working after an initial return.

Future studies should prospectively assess return to work using predefined time points, validated measures of work ability and quality of life, individual-level workplace information, and systematic recording of occupational health involvement and workplace accommodations. Such studies would allow more robust evaluation of the clinical, functional, social, and occupational factors associated with sustainable return to work after colon cancer treatment.

## Conclusions

This retrospective single-centre study describes documented occupational trajectories after adjuvant chemotherapy for colon cancer in working-age patients with active work attachment before diagnosis. Occupational outcomes recorded in routine oncology records were heterogeneous and incompletely captured, including dated return to work, undated return, medical leave, unemployment, disability retirement, insufficient occupational information, and recurrence before return.

The findings support the need for more systematic documentation of occupational status in survivorship care. The exploratory occupational analysis did not establish physical demand or break/restroom autonomy as determinants of return to work. Future prospective studies should use systematic longitudinal follow-up and validated individual-level occupational measures to better understand work ability, workplace conditions, and sustainable occupational reintegration after colon cancer treatment.
